# Industry 4.0-Compliant Occupational Chronic Obstructive Pulmonary Disease Prevention: Literature Review and Future Directions

**DOI:** 10.3390/s24175734

**Published:** 2024-09-04

**Authors:** Zhihao Jiang, Otto Jan Bakker, Paulo JDS Bartolo

**Affiliations:** 1 Faculty of Science & Engineering, The University of Manchester, Manchester M13 9PL, UK; 2 School of Mechanical and Aerospace Engineering, Nanyang Technological University, Singapore 639798, Singapore

**Keywords:** COPD, health monitoring, vital signs monitoring, OHS, wearable sensors, sensor networks

## Abstract

Chronic obstructive pulmonary disease (COPD) is among prevalent occupational diseases, causing early retirement and disabilities. This paper looks into occupational-related COPD prevention and intervention in the workplace for Industry 4.0-compliant occupation health and safety management. The economic burden and other severe problems caused by COPD are introduced. Subsequently, seminal research in relevant areas is reviewed. The prospects and challenges are introduced and discussed based on critical management approaches. An initial design of an Industry 4.0-compliant occupational COPD prevention system is presented at the end.

## 1. Introduction

Occupational diseases, also referred to as industrial diseases, comprise a special category of Occupational Health and Safety (OHS) incidents. Chronic diseases, due to their complicated causes and long-term observation windows, are considered to be the most serious type. Chronic obstructive pulmonary disease (COPD) is a prevailing and typical type of chronic occupational disease. COPD, defined by a progressive and irreversible limitation in airflow in the lungs, is one of the top three fatal diseases [1]. Typically, COPD is caused by a combination of smoking, occupational exposures, environmental pollution, and genetic susceptibility [1]. The Health and Safety Executive (HSE) UK lists 16 occupations linked with a possibly increased chance of getting COPD and 7 substances with the potential to cause COPD (Table 1) [2]. By far, the leading cause of COPD is smoking. As one factor exacerbates the effects of one or more of the other factors, it is impossible to consider them independently [3]. It has been observed that smoking and occupational exposure have comprehensive and even synergistic effects on COPD [4]. If someone works in these industries or is exposed to these substances and is also a smoker, the risk of getting COPD is likely to be increased. Workers that are more susceptible to develop COPD are those that are exposed to substances that can cause COPD. In 2010, three separate surveys reported that the prevalence of COPD in working populations can be as high as 30% [5,6,7]. In Britain, approximately 15% of the 30,000 deaths per year caused by COPD were identified as attributable to the workplace [8]. Therefore, COPD brings a significant economic and social burden, costing the UK National Health Service (NHS) more than GBP 800 million annually [9]. Moreover, it is also responsible for 24 million working days lost per annum in the UK, with an estimated cost of GBP 2.7 billion [9]. In the EU, the total annual direct costs of COPD are EUR 38.6 billion, while in the US, the direct costs are USD 32 billion, with an additional USD 20.4 billion of indirect costs [10,11].

In 2011, a large survey was performed across six different countries (Brazil, China, Germany, Turkey, US, UK), with 2426 participants aged 45–67 years recruited [12]. The results showed that the average retirement age of the participants was around 54 [12]. Early retirement could lead to significant societal and economic repercussions [12]. This is particularly the case where typical state retirement ages are higher, such as in the case of Brazil, Germany, and the UK, where the typical state retirement ages are 65 years and in the US it is 65–67 years, compared to Turkey (58 years for women, 60 years for men) and China (60 years) [12]. COPD not only results in high medical expenses, but might also reduce income sources, as a COPD patient has few job options due to the disability of the lungs [13]. In addition to the current social and economic burdens, it should be noted that there is a plateau effect [14] taking place in the development of new COPD cases in the workplace, meaning there will still be significant burdens in the future. Despite the decrease since 2012, there is still around 85 cases reported every year.

Industry 4.0, which ushers in the digitization of manufacturing, and brings new possibilities for OHS management and success stories on the use of digital technologies for the prevention and intervention of diseases such as post-hepatectomy portal hypertension, musculoskeletal disorders (MSDs), and hand–arm vibration syndrome (HAVS), has been reported [15,16,17]. Digital Twins (DTs) provide a new method of predicting the risk of post-hepatectomy portal hypertension [15], while the Internet of Things (IoT) and machine learning (ML) help in preventing MSDs in workplaces [16] and the HAVS in mechanical harvesting operations [17]. However, little attention has been put on using these technologies in fields like occupational chronic disease management and COPD prevention. It should be noted that the hypothetical scenarios in this review are workplaces where exposure assessments are compulsory for existing COPD hazard, regardless of the percentage of workers who get affected, discussing the feasibility of improving and even replacing traditional exposure assessments and protection methods in workplaces rather than circumventing or advocating existing health and safety legislation, which is there for good reasons. In addition, the main focus is put on the onset of COPD development on individual workers. This review aims to find the answer to the following key research question: what and how does the fourth industrial revolution bring to occupational COPD management? It introduces the current situation of occupational COPD; reviews the latest research in relevant fields such as remote health condition monitoring, wireless working environment control, and ML-assisted assessment and prediction; identifies current knowledge gaps; discusses emerging research opportunities and challenges; and presents recommendations for future works.

## 2. COPD

COPD is a group of lung diseases that make it difficult to breathe. Emphysema and chronic bronchitis are the most common conditions that make up COPD, with a significant number of patients suffering from both conditions to some extent. Emphysema causes alveolar air sacs to become damaged and stiff [18]. Air gets trapped in the alveoli, making it hard to exhale. Chronic bronchitis causes swelling in the airways and an increase in mucus [18]. This excess mucus makes it harder to breathe and leads to a chronic cough. The common symptoms of COPD listed by the NHS include increasing breathlessness, which may only happen when exercising at first, and the patient may sometimes wake up at night feeling breathless; a persistent chesty cough with phlegm that does not go away; frequent chest infections; and persistent wheezing [19]. The symptoms will usually get gradually worse over time, making daily activities increasingly difficult.

### 2.1. COPD Diagnosis

Spirometry is a key method for COPD diagnosis, involving a straightforward test that gauges lung function by assessing the volume of air an individual can exhale forcefully in a single breath. During the test, the forced expiratory volume in 1 s (FEV1) and the forced vital capacity (FVC) of the participant are measured by a spirometer. Typically, a value of FEV1 divided by FVC below 0.7 would be considered to be getting over the threshold value for COPD diagnosis. Spirometry may be performed by a nurse or physician at the GP surgery or a clinic, but it is usually recommended that companies with high COPD risks arrange regular spirometry tests at their facilities for workers [1]. On the other hand, multiple measures are endorsed to ensure that reliable results are obtained. (1) It is advised to avoid smoking for 24 h before the test; (2) the test is normally repeated three times; and (3) application of inhaled bronchodilator medicine is recommended [1,20]. As a result, one test could be time-consuming. According to the NHS, a spirometry test appointment usually lasts around 30 to 90 min [20]. COPD severity can be divided into four stages according to the comparison of the measured FEV1 value to the predicted value based on the participant’s height, weight, and race [1].

Apart from spirometry, there are two other widely used diagnosis methods based on measures of symptoms: the Modified Medical Research Council (mMRC) Dyspnea Scale [21] and the COPD Assessment Test [22], also called the CAT assessment. These methods are based on validated questionnaires to assess COPD in patients according to the symptoms, and do not take into account the FEV1. Regarding the mMRC scale, higher grades denote more severe impacts of COPD on patients’ lives, and studies have shown that a patient’s mMRC score is moderately correlated with pulmonary function measurements and may provide quantitative information complementary to the latter [21]. CAT scores range from 0 to 40, and higher scores indicate higher severity. The foundational study behind the CAT included a cohort of 1503 patients across six countries, demonstrating its effectiveness as a dependable indicator of COPD severity from the standpoint of the patient, regardless of language differences [22].

### 2.2. COPD Indicators and Monitoring

Research on COPD indicators is highly relevant for COPD detection, especially for health hazard assessment of all kinds of COPD risk factors in the workplace. Six relevant indicators were identified:

1. Activities of Daily Living (ADL): COPD is typically accompanied by decreased ADL [23], which makes ADL assessment one of the best COPD evaluation methods. Furthermore, it is vital for COPD patients to increase their ADLs [24]. A study implemented real-time activity classification by placing sensors on the forearm, thigh, and sternum [25]. Wearable technologies such as smart vests and t-shirts were developed to reduce the number of sensors required, and cloud-connected platforms were designed for remote monitoring and interactions [26,27].
2. Volatile Organic Compounds (VOCs): VOCs, such as isoprene and hexadecane, are typical kinds of the COPD biomarkers in exhaled breath [28,29]. A portable spectrometer has been proposed for chemical analysis [30]. However, there are contrary opinions on using VOC profiles for COPD diagnosis. Research shows that VOC profiles could identify patients with COPD accurately [31,32,33], while it was also observed by some studies that VOC profiles cannot distinguish smokers, including former smokers, from COPD patients [34,35].
3. Blood Lactate Level: The blood lactate level is another COPD biomarker [36]. It has been reported that people with COPD tend to have a higher blood lactate level than their healthy counterparts while doing the same activities at the same intensity [36]. As a result, lactic acid has been proposed and used as a biomarker of COPD severity [37]. Several novel approaches using flexible electronics were developed to measure the lactic acid through human tears, saliva, and sweat [38,39,40]. However, in tests, it was found that these electronics were not very comfortable to wear, and their practicality needs further discussion [41].
4. Saliva: Dysphagia is regarded as one of the high-risk phenotypes for the prediction of COPD exacerbation by some studies [42]. Research has found, as a less invasive way to screen dysphagia, that a repetitive saliva swallowing test cut-off value of 5 could be a strong predictor of COPD exacerbation [43]. Compared to bio-samples like blood and sputum, saliva is relatively easy to use, especially for home monitoring. A novel biosensor called COPD saliva-based point-of-care monitor has been designed to enable patients to undergo testing at home and identify exacerbation in time [44,45].
5. Respiration: As aforementioned, the main symptoms of COPD are related to the patient’s respiratory condition. This follows from the medical explanation of why breathing will change is that sternomastoid muscles, which are accessory muscles, are used during the exacerbation period of COPD [46]. Research has explored the consistency and accuracy of breathing sounds at various airflow levels and predetermined bodily sites in individuals with COPD [47]. A conclusion was drawn that the most reliable interval of air flow is 0.4–0.6 L/s, and this applies to respiratory sound parameters at all anatomic locations [47]. Moreover, it is recommended to be considered in computerized auscultation for future use [47]. On the other hand, research shows that the respiration characteristics of COPD differ from other dyspnea diseases [48]. Furthermore, a study found the potential of computerized analyses of respiratory sounds in respiratory status monitoring for people suffering from COPD, and this study achieved “75.8% exacerbations were early detected with an average of 5 ± 1.9 days in advance at [sic] medical attention” [49].
6. Cough and Sputum Production: Chronic cough and sputum production are not only very common in subjects with COPD, but have also been suggested as being predictive of disease progression, exacerbations, and hospitalizations [50]. Research on using chronic cough or phlegm to predict or identify COPD risk severity achieved some success [51,52]. It has been pointed out that each of them is an independent and statistically significant predictor of COPD [51]. Experiments successfully identified a subgroup of participants at a high COPD risk, irrespective of smoking habits. The study contends that the occurrence of a chronic cough or phlegm serves as an early indicator of COPD in a significant number of patients, regardless of their smoking status [51]. Sumner et al. considered cough only in a study, and a comparison was made of 68 randomly selected current and ex-smokers with COPD and smokers without COPD and healthy nonsmokers, using a custom-built cough sound recording device over 24 h. An outcome of this study was that objective cough monitoring is viable. Moreover, it offers a great prospect that cough monitoring can provide timely feedback for COPD interventions and also allows for adapting the new strategy in development [52].

### 2.3. Environmental Risks

Despite various types of jobs or workplaces being exposed to COPD risks (Table 1), the current commonly used protection methods can be broadly divided into three main categories: wet method, e.g., water spray and wet cutting; ventilation, including local exhaust ventilation (LEV) systems and blower fans; and the segregation of workers and dust such as by respiratory protective equipment and a separate control booth. Wet methods use water to capture and remove respirable dust particles from the air. Water spray helps reduce the amount of airborne dust particles, while wet cutting can prevent them from spreading in the first place. However, an investigation showed that the usage of water upsets self-responsibility, since water waste is not considered environment-friendly [53].

Legislation and regulations regarding occupational exposures provide some limitation of values of hazardous substances. The most mentioned are the recommended exposure limit of the National Institute of Occupational Safety and Health (NIOSH) in the US and the Workplace Exposure Limits (WELs) in the UK. The NIOSH also proposed a method to assign COPD substance exposure called COPD-Specific Job Exposure Matrix (JEM), covering eight exposure categories: vapor–gas, sensitizers, fumes, organic dust, mineral dust, combined dust, diesel exhaust, and overall exposure. In a study, the COPD JEM was evaluated by comparison with expert evaluation, and has proved to be a cost-effective generalizable method of assigning occupational exposures [54]. This study also illustrated that COPD JEM has limited sensitivity, and other assessment methods may have higher specificity using more detailed job data [54].

However, there are some significant concerns about the main parts of current OHS management, including diagnosis, protection methods, and risk assessment. First, spirometry tests performed in the workplace could cause a delayed diagnosis due to mis-operation. So far, research has found that more significant drops in FEV1 are required to identify an occupational COPD patient, and the Pulmonary Function Test (PFT) for workers on a regular basis barely helps because of low measurement accuracy [55,56]. Secondly, research shows that current protection methods might not be as effective as they should be. Research conducted among adults aged 40 years and over in China revealed that 46.3% were highly exposed to dust and/or noxious gases and a there was a relatively low rate of protection measures taken, at 26.7%, including dust-proof face covers and gas masks [57]. The analytical results of an investigation in the construction industry indicated that respiratory protection commonly used on construction sites, LEV systems, blower fans, and wet methods were often inadequate systems for the level of exposure encountered [58]. Similar issues arise in underground coal mines due to the ineffectiveness of both group and individual protective measures against dust that is harmful to health, as well as the need to improve both the personal and environmental protection measures [59]. Finally, the COPD risk assessment based on statistics could lead to some omissions. Risk evaluations are often performed en masse, targeting employee clusters facing similar hazardous substances [60]. Such evaluations are executed once per group, leading to findings that represent a mean risk level, rather than pinpointing the risk faced by an individual employee engaged in a particular activity [60]. To sum up, the current management procedure may exhibit the following problems: delayed diagnosis in the potential COPD worker identification, low efficient protection methods, and low accurate exposure assessment caused by a lack of personalization.

## 3. Industry 4.0-Compliant Management

### 3.1. IoT and Data Analysis

The digitalization of the industry brings enormous changes and accelerates the technology revolution in many fields of OHS. For example, digital biomarker monitoring is enabled by means of portable, wearable, implantable, or digestible devices [61]. Digital biomarkers are characterized as measurable indicators of physiological and behavioral states [61]. On the other hand, there is also an increasing trend of using digital technology for healthcare [62,63,64]. A survey in 2015 showed that over 97% of COPD patients use a mobile phone to help manage disease conditions and feel highly satisfied, and 94% of them even showed better treatment compliance [62]. It is forecasted that by 2032, the UK market for wearable technology will soar to an impressive USD 231 billion, showcasing the vast potential of this rapidly expanding industry [63]. In 2022, the worldwide market for wearable technology was estimated to be at USD 61.30 billion and is projected to grow at a compound annual growth rate of 14.6% from 2023 to 2030 [64]. 

Another emerging digitalization and visualization tool for OHS management is the Environment, Health and Safety (EHS) software. It usually assists in monitoring regulatory compliance, enterprise risk, and corporate sustainability data and activities [65,66]. EHS software features are designed to aid with the following [65]:

1. Adherence to and staying current with the latest regulations or directives;
2. Protection of workers by identifying workplace hazards and near misses;
3. Monitoring of training activities of employees on important EHS policies and procedures;
4. Improvement of employee exposure information.

However, EHS software is designed for generalized OHS incidents, while the digitalization and visualization of COPD-related management involves personal health condition evaluation and disease development predication, which requires targeted physiological index monitoring, as aforementioned in Section 2.2. Measurement variables and sensors are more specifically selected to detect changes in respiratory capability, and also the outcomes are much further processed by ML. Reported efforts for COPD management broadly fall into three categories:

1. Remote Health Condition Monitoring: Applying IoT for medical purposes, such as using wearable sensors for wireless monitoring, is a trending topic. A design of the Internet of Medical Things (IoMT) for remote respiratory rate monitoring of COPD patients was proposed to improve doctor–patient communication [67]. It used message queue telemetry transport protocol and could send clinical alarms according to configurable thresholds. Additionally, a smart vest was designed for breathing monitoring during the rest period, with embedded capacitive sensors [67]. Similarly, to make the vest more comfortable to wear, inkjet-printed sensor technology was used [68]. It is stretchable and wearable, and the design achieved high measurement accuracy at different postures and among different patients. In the research of these two smart vests, the only parameter considered was the breath rate of the wearer, and the measurements were supposed to be taken during the rest period. For the same purpose of remote respiration monitoring, much more physiological parameters were covered in the design of a smart mask proposed by Tipparaju et al. [69]. In this work, principle component analysis (PCA) was applied to analyze the respiration pattern of each participant, but neither the pathology basis nor how to use it for a particular disease was considered [69].
2. Working Environment Control: To the best knowledge of the authors, universal examples of Industry 4.0-compliant environmental COPD risk control do not exist, probably due to the large variety of COPD substances. However, research on occupational exposure management IoT systems has been reported [70,71]. A project aimed at sustainable health management presented an IoT-based indoor environment monitoring system tracking O3 concentrations near photocopy machines [70]. The developed sensing node contains a Bluetooth module and a semiconductor O3 sensor, apart from which, the developed IoT system also includes gateway nodes and processing nodes. It was claimed that the design can be expanded to cover larger areas and more pollutants such as hydrocarbons and different-size particles [70]. Similarly, Fathallah et al. conducted work on occupational exposure estimation and proposed a real-time occupational exposure monitoring model [71]. It successfully quantified indoor worker exposure to formaldehyde and CO2 in real time using multi-pollutant sensor nodes and an indoor positioning system [71].
3. ML-Assisted Assessment and Prediction: The introduction of ML to assist diagnosis is a new trend. Zarrin and Wenger developed an Artificial Neural Network (ANN) model for pattern recognition for COPD diagnosis [72]. In this study, eight fundamental parameters were considered: the viscosity of saliva samples, the ambient temperature, patient smoking background, cytokine level, pathogen load, mucin combinations, gender, and age. Moreover, the output was set to four different kinds of disease statuses: healthy, low probability, high probability, and COPD-diseased. After comparing to the actual states, an accuracy rate of 112 out of 200 was achieved [72]. Attempts of COPD readmission prediction have also been made. COPD patients were required to use accelerometer-based wrist-worn wearable devices during daily living and readmission risks for 30 days, and were predicted based on their physical activity, including the activity index and regularity index, and quality of activity [73]. The results from 16 COPD patients showed a sensitivity of 63% and a positive prediction rate of 37.78%, which can be considered a significant improvement in comparison to other clinical assessments [73].

### 3.2. Industry 4.0-Compliant Prevention Based on Underpinning OHS and Medical Management Approaches

A fundamental and well-known approach of OHS management is the hierarchy of controls [74]. This is a system used for the protection of workers from exposure to occupational hazards, and comprises five levels and ranges from the most effective to the least effective [74]:

1. Elimination: physical removal of the hazard;
2. Substitution: replacement of the hazard;
3. Engineering Controls: isolation of people from the hazard;
4. Administrative Controls: change the way people work;
5. Personal Protective Equipment (PPE): protects the worker.

The hierarchy of controls is often used to decide what to do and how to improve the effectiveness of protection. In this concept, elimination and substitution are considered the most effective control methods. However, they may also be the most challenging to implement due to the associated costs and technological requirements [74]. Therefore, engineering and administrative controls, and PPE remain the measure of choice for existing industrial environments. Additionally, when the novel Industry 4.0 solutions discussed here were analyzed and classified, they appeared to fall under the latter three categories of control measures. For instance, this can be seen in the positive outcome from studies [16,75], where introducing IoT and ML in occupational MSD prevention [16] and developing a real-time risk alert IoT system for the underground mining industry [75] also fall into these three aspects.

Engineering controls are preferred over administrative control or PPE because they cut the contact of hazards and workers so that there will be no exposures [74]. At this level, supportive Industry 4.0 technologies such as Wireless Sensor Network (WSN) and IoT have been investigated, with relevant examples being a WSN-based real-time risk alert IoT system for the underground mining industry [75] and an IoT-based system for real-time occupational exposure monitoring [71]. Digital technologies such as ML and augmented reality are also relevant technologies to assist administrative controls. For instance, the ML-enabled ergonomic risk assessment system developed by Low et al. helps employers deal with large data and provides a better understanding to make more feasible and sensible decisions [16]. “Smart” PPE, PPE embedded with digital technologies such as sensors and wireless communication modules, and wearable technologies have received considerable attention [16,75]. They represent a relevant solution for OHS management and play an essential part in the implementation of active protection systems, being effective and allowing for personalization.

As COPD is a chronic disease that cannot be cured, patients require a long-term disease management program. From this aspect, medical management has also attracted digitalization effort. The term Predictive, Personalized, Preventative and Participatory (P4) medicine was coined by Hood and Friend [76], and it is based on the following pillars:

1. The redefinition of medicine as an informative science;
2. The interconnected domains composing complex diseases;
3. The emerging technologies allowing for different approaches to understand and access patient data;
4. New and powerful analytical systems.

The P4 medicine paradigm confirms and points out the features of the new trend of digital healthcare, and many IoT solutions for COPD and other therapies are closely aligned with this paradigm [15,67,69,72]. A relevant example is the use of DT technology and mathematical models of the entire blood circulation used for decision-making of surgical procedures [15]. Moreover, the smart mask for respiratory monitoring developed by Tipparaju et al. [69], and the use of ANN models for COPD diagnosis by Zarrin and Wenger [72] are good examples of emerging technologies used to understand and access patient data, which can collect extensive data and build powerful analytical systems for decision-making.

## 4. Discussion

### 4.1. Identification of Opportunities and Challenges

The applications of emerging digital technologies to OHS management for COPD bring not only opportunities, but also research challenges that must be considered for the further development of this field.

1. Health Condition Detection: current research on how digital technologies such as IoT and artificial intelligence can specially support the treatment of occupational-related COPD, rather than OHS management, more related to protection against and prevention of COPD. Systematic reviews reported that digital health interventions (DHIs) for COPD show some uptake problems, like low compliance rates and lack of personalization [77,78]. Some remote monitoring systems also present restricted utilization to specific times during the day [77]. Moreover, measurements should also be more adjustable to the requirements of the target population [78].
2. Protection: active protection is one of the new trends in OHS 4.0; digital technologies like smart PPE and WSNs can provide more sources and types of data to support further analysis. However, COPD risk factors found in workplaces usually vary. Therefore, monitoring systems with fixed alarm values of one or two substances are not effective enough. It should be noted that some exposure exceeding critical values could be easily avoided by combining environmental monitoring systems with primary real-time intervention control, such as connecting traditional protection equipment like LEV systems and environmental sensors to cyber–physical systems (CPSs), allowing for reducing the exposure level in the workplace and maintaining it under WEL in real time. Regarding personal protection, Adjiski et al. devised smart underground mining PPE by introducing sensors and wireless communication modules into safety wear [75].
3. Assessment: the integration of traditional assessment methods and ML algorithms can improve accuracy and help optimizing management. COPD risk assessment in workplaces needs to be more personalized and dynamic. The lack of personalization of current approaches, which use the same standard for different workers at different ages and different jobs can result in misdiagnoses. With the idea of new conceptual OHS management, digital technologies such as data fusion (e.g., sound and temperature) and ML show high potential for assessment assistance and decision optimization. For example, without motion working state recognition, health condition monitoring could be meaningless. Moreover, unlike other industrial diseases, such as HAVS and MSDs, there are no “ergonomic tools” for COPD, nor well-developed analysis and assessment standards. In addition, it is hard to diagnose or predict COPD as it is a “chronic” disease, influenced by several factors, including various substances and lifestyle habits like exercise and smoking.

The concept of Industry 4.0 and the use of relevant digital technologies will have a significant impact on managing occupational COPD. Smart PPE and Smart Working Environment (SWE) can provide personalized health detection and real-time environmental monitoring, respectively. Then, ML tools will send back optimized orders based on acquired data. Finally, interventions will be performed effectively. However, there are three main challenges that must be considered. Firstly, an OHS management system is unable to evaluate the influence of COPD risk factors on workers timely without continuous personal health condition monitoring. Secondly, commonly used protection equipment and intervention methods are not effective due to a lack of real-time feedback of their effects on the working environment as well as workers. Finally, personalized individual risk assessment for each worker cannot be carried out without knowing which risk factors the worker is exposed to and for how long.

### 4.2. Future Trends and a Vision of Implementation

**Figure 1:**
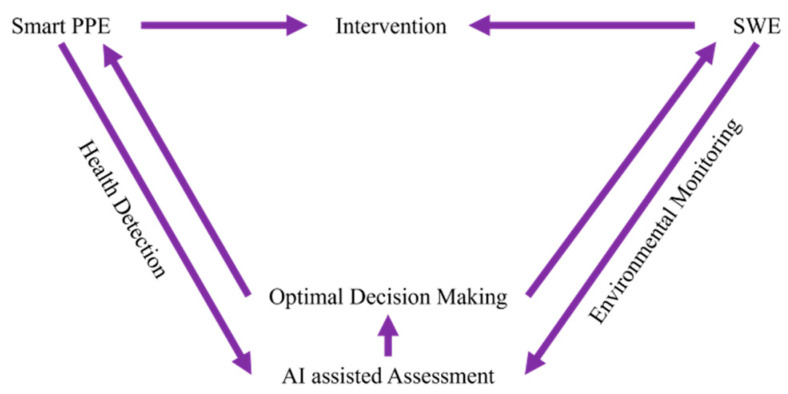
Roles of Industry 4.0 technologies in COPD OHS management.

One of the objectives of this review is to envisage a conceptual framework for Industry 4.0-compliant occupational COPD management. Therefore, relevant supporting technologies were identified and discussed. Based on the literature review and the identified research gaps and challenges, a suitable occupational COPD management framework was envisaged, as shown in Figure 1. In this framework, smart PPE, using wearable technologies, allows for the continuous monitoring of workers’ health conditions in the workplace. The WSN in the SWE manage environmental data acquisition with advanced analysis being performed by ML. Finally, the optimized outcomes are sent as intervention instructions through CPS.

Based on the analysis presented in Section 3, the development of the envisaged framework requires further research and development in the following three main domains:

1. Real-Time Monitoring: Continuous health condition monitoring is necessary to analyze the influences of COPD risk factors on workers. Motion monitoring is also needed for working state recognition, while real-time environmental monitoring helps identify the COPD risk factors and workers’ positions.
2. Dynamic Exposure Assessment: Data fusion technology can combine the information acquired from the wearable sensors and environmental sensors, using it to establish what happened where, when, and to whom. Exposure assessments like exposure profiles and hot points should be carried out considering job types and layout.
3. Effective and Targeted Intervention: Intervention must be performed in a personalized way. Instead of pre-setting values, ML algorithms can help producing effective and targeted intervention standards based on different health conditions and jobs. For example, intervention including alarm, LEV control, and smoke cessation suggestions could be delivered through CPS in the workplace. This way, the lung function prediction of each worker and COPD risk assessment of a workplace could be performed without long-time observations.

Cybersecurity and privacy must also be addressed, as they might affect workers’ willingness to take part in a digitalized OHS management process. Cybersecurity plays a vital role in protecting workers’ privacy from cyberattacks [79]. Recently, a study showed that the majority of workers have common concerns about data sharing, being obligatory to explain the type and usage of data acquired to the workers [80]. Otherwise, workers may resist to accept the use of digital technologies due to concerns of personal information being used by third parties [81]. With the public’s rising concerns about privacy and security, efforts have been made to enhance data protection; research focusing on CPS cybersecurity listed out several challenges such as reduced performance and operational security delays, and possible problem sources like remote access and physical damage [82]. Another reason why workers might refuse to participate in IoT-based occupational COPD management is that personal assessment results and targeted intervention suggestions may make them feel self-blamed and discouraged, especially smokers [83]. The need for an effective attack and anomaly alert system has also been emphasized within IoT-based environments due to the security risks posed by unauthorized access, attacks, and anomalies. The complexity of IoT environments, combined with the sensitivity of the data they process, demands robust detection mechanisms that can operate in real time without draining power or requiring significant memory resources [84].

**Figure 2:**
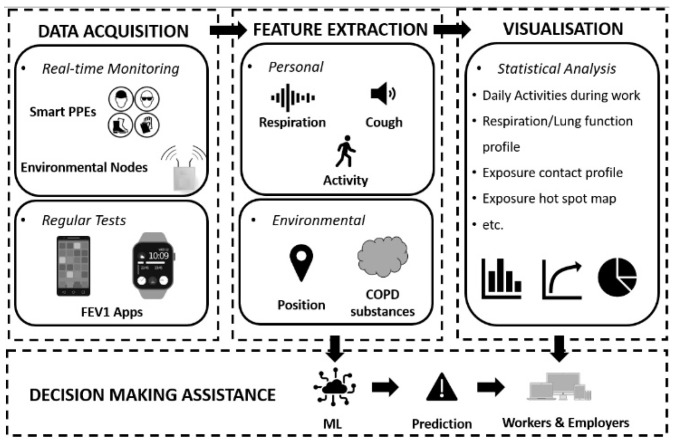
The proposed occupational COPD management workflow diagram.

Based on this literature review, a recommended workflow diagram for Industry 4.0-based occupational COPD management in the workplace was proposed (Figure 2). The proposed methodology comprises four main steps, as shown in Figure 2. The first step corresponds to data acquisition. In this case, data are collected from wearable and environmental sensors. Data can also be collected through spirometry tests, and several apps have been developed for this [79,85,86]. Tests that may require interrupting production can be performed post-work shift. The second part corresponds to feature extraction. In this case, information about personal health conditions and the working environment is extracted through data processing. The outputs of this step can be used as the inputs of ML (e.g., an ANN, to give a prediction or indication of someone’s COPD risk severity or that of the working environment) and for statistical analysis. The visualization step (third step) generates relevant graphs, such as the lung function profile, exposure contact profile, and exposure hot spot map based on data analysis. These graphs, together with the other outputs from ML, could heighten workers’ and employers’ awareness, help communicate COPD risks in better ways, and assist decision-making (fourth step) to achieve more effective prevention and intervention. As the final outputs, this system aims to provide two different kinds of information: the visualized statistical data and ML-generated suggestions. In detail, the daily activities during work, along with the respiration/lung function, could help workers know their health conditions better and raise concerns for their own health; the exposure contact profile and the exposure hot spot map can enable efficient management improvement such as shift arrangements and the usage of LEV systems. Limited by interpretability and explainability, the two primary roles of ML assistance, for now, are to implore workers to seek for further medical examinations and employers to perform workplace exposure assessments. In future work, the potential of this system to provide personalized granular monitoring to assist medical diagnosis can be further discussed with efforts in interpretability such as medical explanations from experts in a relative field and medical mathematic models of lungs.

Additional information on relevant variables considered by the proposed methodology and corresponding sensors are presented in Table 2. The spirometry test is an essential part of lung function monitoring, even though it cannot be performed in real time or without interpreting the work. Therefore, data of workers’ FEV1 and FVC must be acquired either through apps on smart devices or any other types of portable devices. Based on the review of COPD monitoring, one of the sensible ways to help identify COPD in advance is to record and analyze respiration and coughs. Additionally, these two parameters show some potential to shorten the observation period, which typically takes more than one year to identify a worker who is at a high COPD risk when only the health conditions (oxygen saturation, heart rate, breath rate) are monitored [1,55]. On the other hand, real-time respiration monitoring makes the activity recognition a compulsory part to differentiate the respiration recordings of different gaits, such as working, walking, and sitting. The worker’s position information is needed for exposure maps and a personal exposure contact profile according to each worker’s routine in the workplace. Environmental COPD substance monitoring could highly vary for different workplaces. The construction industry and other workplaces with respirable COPD substance particles form areas of priority, as they are significant contributors to occupational COPD [87].

## 5. Conclusions

In conclusion, COPD is a highly work-related health problem. It directly causes a large healthcare burden and workforce loss due to earlier retirement and disability. Traditional COPD management focuses on the legislation of exposure limits and regulation of protection procedures, which are carried out manually by periodic checks. This paper reviews and compares emerging technologies, such as wearable sensors for health condition detection, WSN for environmental monitoring, and ML for severity evaluation, that enable timely and personalized data acquisition, and optimized and targeted intervention. The literature survey of cutting-edge research in relevant fields has found that Industry 4.0 technologies, including wearable technology, WSN, IoT, CPS, and ML, allow for observing and understanding both COPD and OHS in a whole new way, and have the potential for breaking the plateau effect on the current occupational COPD management. The identified knowledge gaps are as follows:

1. Heath condition detection methods from industrial perspective are needed for the purpose of occupational protection. Moreover, for different target populations, measurements should be adjusted for a better uptake.
2. Traditional hazard assessments rely on manual periodic checks, which are both time-consuming and expensive, and lead to less accurate results. Sensor-based hazard monitoring is supposed to deal with a wide range of hazards. Dynamic WELs, i.e., exposure thresholds varying with time, should be calculated and derived to drive active protection or real-time intervention control introduced by CPS.
3. COPD is a chronic disease with complex causes and varies from person to person. Compared to other diseases, it is difficult to convert current COPD diagnosis criteria into computer algorithms. A personalized diagnosis taking an individual’s physical states and circumstances into account is vital for accurate conclusion in decision making.

The roles that Industry 4.0 technologies can play in COPD OHS management are suggested in Figure 1, illustrating challenges in real-time monitoring, dynamic exposure assessment, and effective and targeted intervention. In addition, this paper presents a new Industry 4.0-compliant solution (Figure 2) based on wearable technology, IoT, and ML, which allows for digitalized and visualized personal health condition and exposure–contact monitoring, and provides risk evaluation and prediction. Its key advantages are non-disruptive monitoring, personal exposure tracking, and multi-feature driven analysis. By reviewing related work, the relevant indictors for monitoring, standards to refer to, and possible sensor selection are also presented in Table 2 based on the proposed solution. The limitations of this research relate to the privacy-sensitive information and the explainability of the ML-generated results. Features that involve personal health information must be handled with care to protect privacy. When it comes to IoT developed for practical use, cybersecurity will have an important part. Moreover, ML results could be questioned for interpretability and explainability, and confidence in the model will grow when the medical understanding of COPD diagnosis methods, other than those currently used, also grows. This solution must be cost-effective and reliable, and must also guarantee participants’ information security. The utilization of unlabeled and anomaly samples is also worthy of consideration [91].

## Figures and Tables

**Table 1:** Occupations and substances highly relevant to COPD [2].

Occupation		Substance
Agriculture	Brick making	Cadmium dust
Construction	Dock workers	Organic dusts
Textiles	Quarries	Grain and flour dust
Mining	Welders	Welding fumes
Stonemasonry	Cadmium	Cadmium fumes
Rubber	Plastics	Silica dust
Petroleum workers	Foundry workers	Mineral dust
Flour and grain workers in the food industry		

**Table 2:** Key variables and possible sensors.

Variable	Standard	Indication	Sensor
FEV1	15% or 500 mL decline in one year [55]	COPD alarm	Portable spirometer [79,85,86]
Respiration rate	25 breaths per min (bpm) [85,88]	COPD exacerbation	Acoustic sensor [88]
Cough	3 months per year for 2 years [89]	Chronic bronchitis	Microphone [90]
Activity	N/A	Working, walking, or sitting	Accelerometer
COPD substance	N/A	COPD substance concentration	Air composition analysis
Position	WELs	Workers’ positions	Wi-Fi, Bluetooth, etc.
